# Study on the Effect of Different Concentrations of SO_2_ on the Volatile Aroma Components of ‘Beibinghong’ Ice Wine

**DOI:** 10.3390/foods13081247

**Published:** 2024-04-19

**Authors:** Baoxiang Zhang, Weiyu Cao, Changyu Li, Yingxue Liu, Zihao Zhao, Hongyan Qin, Shutian Fan, Peilei Xu, Yiming Yang, Wenpeng Lu

**Affiliations:** 1 Institute of Special Animal and Plant Sciences of Chinese Academy of Agricultural Sciences, Changchun 130112, China; zbx0319@126.com (B.Z.); 82101202231@caas.cn (W.C.); lichangyu@caas.cn (C.L.); liuyingxue@caas.cn (Y.L.); qinhongyan@caas.cn (H.Q.); fanshutian@caas.cn (S.F.); xupeilei@caas.cn (P.X.); yangyiming@caas.cn (Y.Y.); 2 School of Foreign Languages, Jilin Science and Technology Vocational College, Changchun 130123, China; 13504486086@163.com

**Keywords:** HS-GC-IMS, volatile components, OAV value, VIP value, sensory evaluation

## Abstract

SO_2_ plays an important role in wine fermentation, and its effects on wine aroma are complex and diverse. In order to investigate the effects of different SO_2_ additions on the fermentation process, quality, and flavor of ‘Beibinghong’ ice wine, we fermented ‘Beibinghong’ picked in 2019. We examined the fermentation rate, basic physicochemical properties, and volatile aroma compound concentrations of ‘Beibinghong’ ice wine under different SO_2_ additions and constructed a fingerprint of volatile compounds in ice wine. The results showed that 44 typical volatile compounds in ‘Beibinghong’ ice wine were identified and quantified. The OAV and VIP values were calculated using the threshold values of each volatile compound, and t the effect of SO_2_ on the volatile compounds of ‘Beibinghong’ ice wine might be related to five aroma compounds: ethyl butyrate, ethyl propionate, ethyl 3-methyl butyrate-M, ethyl 3-methyl butyrate-D, and 3-methyl butyraldehyde. Tasting of ‘Beibinghong’ ice wine at different SO_2_ additions revealed that the overall flavor of ‘Beibinghong’ ice wine was the highest at an SO_2_ addition level of 30 mg/L. An SO_2_ addition level of 30 mg/L was the optimal addition level. The results of this study are of great significance for understanding the effect of SO_2_ on the fermentation of ‘Beibinghong’ ice wine.

## 1. Introduction

Ice wine is a sweet wine that is made by fermenting the naturally frozen grape juice from the vines when the temperature drops to −7~−8 °C [1]. Canada and Germany are the leading producers of ice wine, while China, Austria, and the United States also produce ice wine in large quantities. In recent years, the annual production of ice wine in China has reached 300 million liters, especially in Huanren County, Liaoning Province, and the Yalu River Basin, Jilin Province, where the ice wine industry is developing rapidly [2]. The ‘Beibinghong’ grape is the world’s first wild grape variety (*Vitis amurensis Rupr*) that can make ice wine and is very popular in northeast China. It has the advantages of high cold tolerance and stable yield. Compared to unfrozen grapes, frozen grapes contain high concentrations of sugar, aroma, and flavor compounds, giving the resulting ice wine a rich, fruity flavor. After alcoholic fermentation, ice wine still contains a rich concentration of residual sugar, which gives it a solid, sweet flavor. The ‘Beibinghong’ ice wine has a more rounded taste and mellow aroma than the ‘Beibinghong’ dry wine, and ice wine has received a lot of attention because of its unique flavor.

‘Beibinghong’ is one of the famous high-quality grape varieties; *Vitis amurensis Rupr* belong to the East Asian grape family. ‘Beibinghong’ is an interspecific cross between ‘Zuoyouhong’ as the mother and ’84-26-53′—a mountain-European F2 grape variety with low acid and high sugar content, thick skin and large bunches—as the father. This interspecific hybridization from the F5 generation to select and breed a new variety of ice wine brewing was first made in 1995. [3]. The ‘Beibinghong’ is the first *Vitis amurensis Rupr* variety cultivated at home and abroad to produce ice wine, and its preciousness and rarity exceed that of existing varieties on the market. It is cultivated in Inner Mongolia, Shaanxi, Gansu, and Northeastern provinces, with a planting area of about 8600 hm^2^ [4], and it is the first *Vitis amurensis Rupr* variety cultivated at home and abroad to produce ice wine. Its main planting area is in Jilin Province, and it occupies an essential position in cultivating unique plants in Jilin Province [5]. In recent years, ‘Beibinghong’ has been widely cultivated in the “Changbai Mountain” region of Jilin Province and the “Huanren” region of Liaoning Province and is the most popular variety of ice wine. Local breweries develop ice wine products and have distinctive aromas of “sweet”, “honey”, “roasted”, and “caramel”.

Volatile compounds are essential for wine quality, determining the characteristics of specific varieties and reflecting the effects of environmental conditions and viticultural management [6]. SO_2_ is an indispensable additive in the wine-making process. It has the following leading roles in the production and preservation of wine: inhibiting the growth of harmful bacteria and yeasts, eliminating dissolved oxygen, inhibiting polyphenol oxidizing enzymes and the infestation of stray bacteria, protecting the hygienic quality and stability of wines, and the addition of SO_2_ in appropriate quantities can attenuate the undesirable flavor of wines [7,8,9]. It prevents oxidative deterioration of wines and maintains their color, aroma, and flavor. Increases the acidity of wines, improves the balance and freshness of wines, promotes clarification and stabilization of wines, and reduces cloudiness and sedimentation of wines. SO_2_ should also be used in moderation, as excessive SO_2_ can adversely affect the quality of the wine and human health [10], such as reducing the aromatic intensity of the wine and masking its character and terroir, producing irritating odors that affect the taste and enjoyment of the wine. This causes allergic reactions such as headaches, breathing difficulties, and skin rashes, which are inappropriate for some people [11].

The SO_2_ content of wines must be strictly controlled, and each country and region must have its legal regulations and standards. According to EU regulations, the SO_2_ content in red wine should not exceed 150 mg/L, and white and pink wine should not exceed 200 mg/L. In China, according to The national standard GB 2760-2014 [12], SO_2_ ≤ 250 mg/L, and according to The national standard GB 7718-2011 [13], as long as SO_2_ is used in food, it has to be marked on the food label [14]. The overall goal of SO_2_ addition prior to wine consumption is to achieve the desired level of free SO_2_ at the lowest possible total SO_2_ [15]. The amount and timing of the addition of SO_2_ can affect the aroma of the wine in different ways. The right amount of SO_2_ protects the fruity and floral aromas of the wine from oxidative deterioration and increases the complexity and aging potential of the wine. The timing of the addition of SO_2_ is also essential, and in general, the earlier it is added, the more significant the impact on the aroma of the wine. For example, adding SO_2_ before wine fermentation can inhibit the growth of non-winemaking yeasts and maintain the cleanliness and purity of the wine. However, it can also reduce the aromatic diversity and complexity of the wine, and the addition of SO_2_ after wine fermentation can inhibit the growth of lactic acid bacteria and prevent the contamination of acetic acid bacteria. However, it also affects the taste and style of the wine. The study not only analyzed the chemical effects of SO_2_ on flavor substances only at the level of the sensory evaluation but also comprehensively from the point of view of the sensory evaluation, although the use of SO_2_ in winemaking is well known [16,17,18,19,20]. However, few articles have explored its effect on wine flavor in-depth, and even fewer studies have investigated the effect of different SO_2_ additions on the aroma of ‘Beibinghong’ ice wine, a special ice wine variety in the Jilin region. The study of ‘Beibinghong’ ice wine, which is a unique ice wine variety from the Jilin region, can improve the flavor quality of the wine by adjusting the strategy of using SO_2_, assessing the effect of SO_2_ concentration on the flavor of the wine in a more precise way, exploring how SO_2_ affects the fermentation process and aroma characteristics of the wine, and identifying the volatile compounds that play a vital role in the different amounts of SO_2_ added to the wine.

This study measured the fermentation start and end times, basic physicochemical properties, and volatile aroma compounds of ‘Beibinghong’ ice wine harvested in 2019. The fingerprints of volatile compounds of ice wine brewed with different concentrations of SO_2_ were established, and its brewed ice wine was tasted. In the experiment, SO_2_ was added by adding solid potassium metabisulfite (PMS), and each gram of PMS produced 0.56 g of SO_2_, i.e., 10 mg of SO_2_ required 17.86 μg of PMS, which was then added in eight treatments, i.e., 10 mg/L, 20 mg/L, 30 mg/L, 40 mg/L, 50 mg/L, 60 mg/L, 80 mg/L, and 100 mg/L, in the following order. Eight treatments were performed to determine the optimal amount of SO_2_ addition at the same temperature and under the same yeast strain and enzyme treatment conditions.

### 1.1. Materials and Reagents

#### 1.1.1. Experimental Materials

‘Beibinghong’ ice grapes harvested from vineyards of Yujiang Valley Winery Co., Ltd. (Ji’an, China) in Ji’an City for ‘Beibinghong’ ice wine production; yeasts BV818 and CEC01 (Angie’s Yeast Co., Ltd., Yichang, Hubei, China); pectinases RF and RCO (AB Enzymes, Darmstadt, Germany); and potassium metabisulphite (SAS SOFRELAB OENOFRANCE).

#### 1.1.2. Reagents

Analytical purity: sulfuric acid, sodium chloride, potassium chloride, sodium bicarbonate (Beijing Chemical Plant, Beijing, China); tannic acid (Tianjin Guangfu Fine Chemical Research Institute, Tianjin Fine Chemical Research Institute, Tianjin, China); Folin–Denis reagent (US Sigma, St. Louis, MO, USA); anhydrous sodium carbonate (Tianjin Hengxing Chemical Reagent Manufacturing Co., Ltd., Tianjin, China); glacial acetic acid, hydrochloric acid, anhydrous ethanol, sodium hydroxide, phosphoric acid (Beijing Chemical Plant); potassium hydrogen phthalate, anthracene ketone (Sinopharm Chemical Reagent Co., Ltd., Shanghai, China); anhydrous sodium acetate (Shanghai Hubtest Chemical Co., Ltd., Shanghai, China); dextrose (Tianjin Hengxing Chemical Reagent Manufacturing Co., Ltd., Tianjin, China).

Chromatographic purity: methanol (TEDIA Reagents, Fairfield, OH, USA), 4-methyl-2-pentanol (Shanghai Lianshuo Biotechnology Co., Ltd., Shanghai, China).

Fermentation auxiliaries: CEC01 active dry yeast (Angel Yeast Co., Ltd., Yichang, China); potassium metabisulphite (Yantai Dibs Homebrewer Co., Ltd., Yantai, China).

### 1.2. Instruments and Equipment

Agilent High Performance Liquid Chromatograph (Agilent Technologies Ltd., Santa Clara, CA, USA); FlavourSpec^®^ Flavor Analyzer (Shandong Haineng Scientific Instrument Co., Ltd., Zibo, Shandong, China); BSA224S-CW Sartorius Electronic Balance (Sartorius Scientific Instruments Co., Ltd., Göttingen, Germany); PAL-1 Digital Hand-held Refractometer (ATAGO, Tokyo, Japan), CJJ-931 Dual-link Magnetic Heating Stirrer (Jiangsu Jintan Jincheng Guosheng Experimental Instrument Factory, Changzhou, Jiangsu, China); HWS-12 type electric thermostatic water bath (Shanghai Yiheng Scientific Instrument Co., Ltd., Shanghai, China); KQ-300E type ultrasonic cleaner, snowflake ice machine (Beijing Changliu Scientific Instrument Co., Ltd., Beijing, China), FA1004B electronic balance (Shanghai Yue Ping Scientific Instrument Co., Ltd., Shanghai, China), DHG-9240 constant temperature drying oven (Shanghai Yihang Scientific Instrument Co., Ltd., Shanghai, China), WAX chromatography columns (U.S. RESTEK, Bellefonte, PA, USA); Milli-Q Advantage A1 ultrapure water apparatus (Millipore Corporation, USA); Cary60UV-Vis UV spectrophotometer (Agilent Technologies Ltd., Santa Clara, CA, USA).

### 1.3. Methods

#### 1.3.1. Process Flow of Brewing ‘Beibinghong’ Ice Wine

The process of ice wine production involves harvesting the fruit, screening, and de-stemming. The harvested fruit is then granulated, pressed, and preserved by adding different concentrations of SO_2_. Three sets of replicate brewing experiments were conducted using three fermenters per treatment to ensure the reproducibility of the experiments. Gum reduction is carried out, followed by a low-temperature maceration at 2–4 °C. The wine is then post-tempered to 15 °C. Subsequently, the post-temperature was adjusted to 15 °C, and controlled fermentation was carried out with the addition of CECO1 yeast at a dosage of 250 mg/Kg. After completion of fermentation, fermentation was stopped, and crude filtration was carried out to obtain the original wine. After controlled aging, the wine is then fine-filtered and sterilized. After passing quality tests, the final product is bottled, sealed, and labeled as ice wine.

#### 1.3.2. Sample Labeling

The amount of SO_2_ used according to the quality of treated iced grape juice was as follows: sample No. 1 (10 mg/L), sample No. 2 (20 mg/L), sample No. 3 (30 mg/L), sample No. 4 (40 mg/L), sample No. 5 (50 mg/L), sample No. 6 (60 mg/L), sample No. 7 (80 mg/L), sample No. 8 (100 mg/L).

#### 1.3.3. Detection Methods of Basic Physical and Chemical Indexes

Soluble solids were determined by handheld refractometer, and titrable acid content of wine was determined by indicator method according to The GB/T 15038-2006 [21]. The alcohol content is measured by the alcohol meter method. The total sugar content in wine was determined by anthrone and sulfuric acid colorimetric method, and standard koji was prepared by standard glucose solution. The total anthocyanin content in grape juice was determined by pH difference method, i.e., anthocyanin reacted with potassium chloride buffer (0.025 M, pH = 1) and acetic acid buffer (0.4 M, pH = 4.5), and then the difference of 520 nm and 700 nm was calculated. Total phenol content—Folin–Ciocalteu colorimetric method. Dry extract content: refer to the dry extract test method in the national standard (GB/T 15038-2006).

#### 1.3.4. Quantification of Volatile Compounds in ‘Beibinghong’ Ice Wine by HS-GC-IMS

Headspace-gas chromatography-ion migration spectrometry (HS-GC-IMS) determined volatile substances in wine. The FlavourSpec^®^ flavor analyzer was used to take a 1 mL sample, place it into a 20 mL headspace bottle, add 20 ppm 4-methyl-2-amyl alcohol 10 μL, incubate at 60 °C for 15 min, and then inject it into the sample.

Chromatographic conditions: the column was WAX column (15 m × 0.53 mm,1 µm), column temperature was 60 °C, carrier gas was N2, IMS temperature was 45 °C, and chromatographic conditions were shown in Table 1.

The conditions of automatic headspace injection were as follows: injection volume of 100 μL, incubation time of 10 min, incubation temperature of 60 °C, injection needle temperature of 65 °C, and incubation speed of 500 rpm; 4-methyl-2-pentanol was used as the internal standard for the analysis, and the concentration of 198 ppb, the signal peak volume of 493.34, and the intensity of each signal peak was about 0.401 ppb.

Quantitative calculation formula:

$$Ci=\frac{Cis*Ai}{Ais}$$

*Ci* is the calculated mass concentration of any component in µg/L, *Cis* is the mass concentration of the internal standard used in µg/L, and *Ai*/*AIS* is the volume ratio of any signal peak to the signal peak of the internal standard. The NIST database and IMS database are built into the software for the qualitative analysis of the substances.

#### 1.3.5. Odor Activity Value (OAV) Calculation

OAV was used to assess the contribution of volatile compounds to the overall aroma of the wine. The concentration of volatile compounds was divided by the odor threshold (OT) to calculate the OAV value. Volatile compounds with OAV > 1 were considered to be types of aroma-active compounds, and the larger the OAV value, the more significant the contribution of components to the flavor; the OAV value can help to determine the critical aroma substances in food or plants [22,23,24], analyze the causes of flavor differences, and optimize the flavor quality of wine aroma characteristics formation plays an important role.

### 1.4. Sensory Evaluation

Sensory evaluation methodology: the wines were subjected to quantitative descriptive analysis (QDA) by a trained sensory panel of 17 tasters (10 women and 7 men, aged 24 to 52 years, with an average of 33 years). These experts were recruited based on their motivation and availability, having been trained according to the national standards ISO 6658 [25] and ISO 8586 [26] prior to the sensory evaluation. According to the definitions in the published literature, according to the definitions in The national standard GB 15038-2006 [27,28], and based on the discussion results, specify the development of a sensory evaluation form (Table 2). The samples were marked with three numbers and submitted to the tasters randomly.

### 1.5. Statistical Analysis of Data

Excel 2010 was used to organize the experimental data statistically, and an analysis of variance (ANOVA) was performed using SPSS (version 22.0, IBM, Armonk, NY, USA). Statistical). Statistical analyses were performed on the experimental data to check for significant differences in the individual results, and all the data were expressed as mean ± standard deviation. Differences between the two groups were considered significant at *p* < 0.05. Simca 14.1 software was used for OPLS-DA and VIP value analysis; GC-IMS assay was done with Savitzky Golay for smoothing and denoising, and migration time normalization was done by setting the RIP position as 1, i.e., dividing the actual migration time by the peak out time of the RIP to obtain the approximate migration time. The Reporter plug-in was used to directly compare the spectral differences between the samples, and the Gallery Plot plug-in was used to compare the fingerprints visually and quantitatively to compare the differences of VOCs between different samples. Heat map and correlation analysis were performed using the OmicShare tools, a free online platform for data analysis (https://www.omicshare.com/tools (accessed on 27 July 2020)).

## 2. Results and Analysis

### 2.1. Fermentation Process and Basic Indexes of ‘Beibinghong’ Ice Wine with Different SO_2_ Additions

The yeast treatment time in this experiment was 11 December 2019, and the initial solid content of ‘Beibinghong’ grape juice was 40.3%. It can be seen from Table 3 that the fermentation time was 24 h at 10 mg/L and 48 h at 30, 40, and 50 mg/L. The fermentation time of 10 mg/L, 40 mg/L, and 50 mg/L was 23, 23, and 21 days, but the red wine was suitable for slow fermentation at low temperatures. This is in line with the findings of Sun Hening and others [29] that the addition of SO_2_ prior to fermentation inhibits yeast activity in the short term and increases the delay in yeast multiplication, leading to a delay in fermentation; however, during this period, it allows the must to remain static and encourages the precipitation of impurities, colloidal substances, and decomposed tartaric acid, which is highly susceptible to the formation of tartar in wines. Wang et al. [30] studied the effect of SO_2_ on the fermentation process and quality of pineapple wine and found that SO_2_ had a significant effect on the time of starting fermentation when the concentration of SO_2_ was 150 mg/L or less, the time of starting fermentation was around ten h. With the increasing concentration of SO_2_, the time of starting fermentation was delayed significantly, and when the concentration of SO_2_ was 250 mg/L, the time of starting the fermentation time was more than 3 d at a concentration of 250 mg/L, which was the same as our results, indicating that the higher concentration of SO_2_ affects the fermentation of fruit wines. The fermentation time of fruit wines should not be too long, so it should be considered comprehensively. The total sugar of ice wine fermented with different amounts of SO_2_ was found to be above 160.0 g/L, which indicates that different amounts of SO_2_ do not have much effect on the total sugar, which is the same as the results of the study by Mou Jingxia et al. [10]. The total acid content of SO_2_ was relatively low at 10 mg/L, 20 mg/L, 30 mg/L, 40 mg/L. The level of dry leachate index is closely related to the raw materials, production process, and storage method of wine, and it is one of the essential symbols of the quality of wine [31]. Dry leachate was higher at SO_2_ additions of 30 mg/L, 40 mg/L; From the above table, adding 30 mg/L and 40 mg/L SO_2_ is more appropriate.

### 2.2. Changes in Anthocyanin Content of ‘Beibinghong’ Ice Wine Brewed with Different SO_2_ Additions

Color is one of the most critical indicators affecting the sensory quality of red wine, and anthocyanin, a class of flavonoid compounds with a benzopyran structure, is an essential water-soluble natural pigment in red wine, as well as a crucial color-presenting substance, with a variety of critical physiological functions and biological activities [32,33]. During grape growth and development, anthocyanosides are biosynthesised via the phenylpropane-flavonoid pathway. In wine, there is an equilibrium between the various states of anthocyanins, and their color expression is closely related to the structure and morphology of the anthocyanin molecule [34]. After human consumption of wine, wine anthocyanosides are mainly metabolized and absorbed by intestinal flora in the colon. The type, state, and content of anthocyanosides are essential in red wines’ color characteristics and aging potential. Changes in the content of anthocyanosides in ‘Beibinghong’ ice wine brewed with different SO_2_ additions are shown in Table 3.

As shown in Table 4, the content of anthocyanin was higher when SO_2_ was added at 30 mg/L, 60 mg/L, and 80 mg/L, especially at 80 mg/L, which reached the highest value. However, the total acid content was slightly higher because ‘Beibinghong’ belongs to the *Vitis amurensis Rupr* variety. From the taste and quality perspective, the SO_2_ addition was not too high, so 30 and 60 mg/L were more appropriate. A moderate addition of SO_2_ has a protective effect on the anthocyanins in wine [35]. SO_2_ inhibits the action of oxidative enzymes and prevents oxidation of the raw material, which helps to maintain the stability and color vividness of the anthocyanosides. The addition of SO_2_ also helps to select the fermentation microorganisms, clarify the fermentation matrix, and regulate the acidity of the fermentation matrix, which indirectly affects the solubilization and stability of the anthocyanosides [36,37,38,39]. J Bakker et al. [40] found that as the level of SO_2_ increased during winemaking, more anthocyanins were extracted. During maturation, all wines lost color and increased brownness. Wines without added SO2 browned more severely than wines with added SO_2_. This also shows that SO2 is essential to maintain color stability in wine production.

However, when the added SO_2_ level is too high, it can adversely affect anthocyanin [41]. This is in line with our findings that an addition of sulphur dioxide that is too high affects the content of anthocyanosides. High concentrations of SO_2_ may generate sulfites in acidic environments, which are capable of reacting with anthocyanoside molecules, leading to the formation of anthocyanoside sulfites, which are colorless, and therefore reduce the anthocyanoside content of wines, thus affecting the color of the wines. SO_2_ has both protective and potentially damaging effects on the anthocyanosides in wine, depending on the amount added and the conditions of use. The amount of SO_2_ added during the winemaking process needs to be precisely controlled to maximize the positive effects and minimize the negative effects on the anthocyanins, thus ensuring optimal wine quality and taste.

### 2.3. HS-GC-IMS Analysis of ‘Beibinghong’ Ice Wine Brewed with Different SO_2_ Additions

#### Fingerprints of Volatile Components of ‘Beibinghong’ Ice Wine Brewed with Different SO_2_ Additives

**Figure 1:**
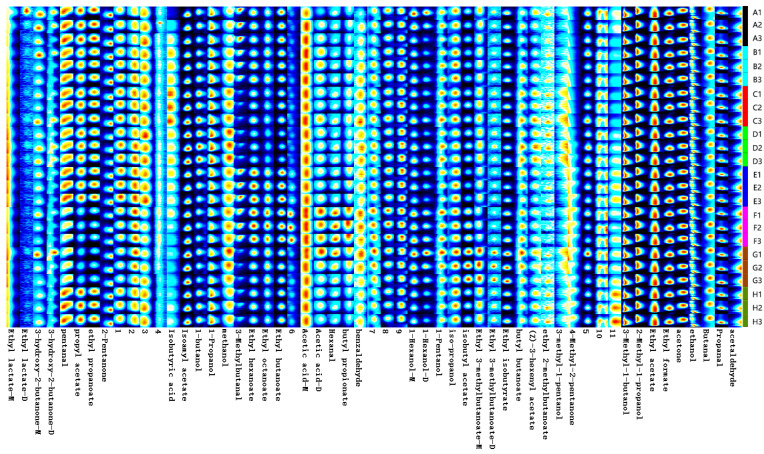
Fingerprints of volatile compounds of ‘Beibinghong’ ice wine under different treatments (Note: A–H are the amounts of SO_2_ added, in the order of 10 mg/L, 20 mg/L, 30 mg/L, 40 mg/L, 50 mg/L, 60 mg/L, 80 mg/L, and 100 mg/L. Same below).

In order to analyze the variability of volatile substances in ice wine brewed with different concentrations of SO_2_, we constructed a fingerprint of volatile flavor compounds based on all the signal peaks in the two-dimensional HS-GC-IMS spectra (Figure 1). Each sample was measured three times in parallel, and the darker color indicated a greater peak intensity and higher content. The composition and differences of volatile flavor compounds in ice wine brewed with different concentrations of SO_2_ were revealed from the fingerprints.

**Figure 2:**
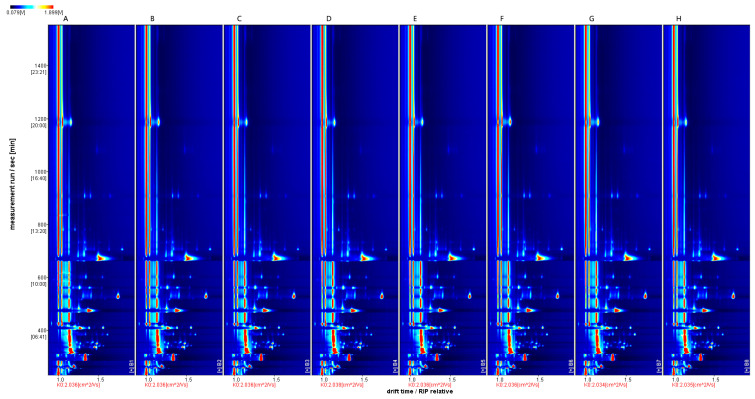
HS-GC-IMS 2D spectrum (top view). Note: The background of the whole graph is blue, and the red vertical line at horizontal coordinate 1.0 is the RIP peak (reactive ion peak, normalized). The vertical coordinate represents the retention time (s) of the gas chromatogram, and the horizontal coordinate represents the ion migration time (normalized). Each point on both sides of the RIP peak represents a volatile organic compound.

As shown in Figure 2, the volatile compounds in the eight wine samples were well separated, and the aroma fingerprints of the wine samples differed significantly, mainly in the content of volatile compounds.

**Figure 3:**
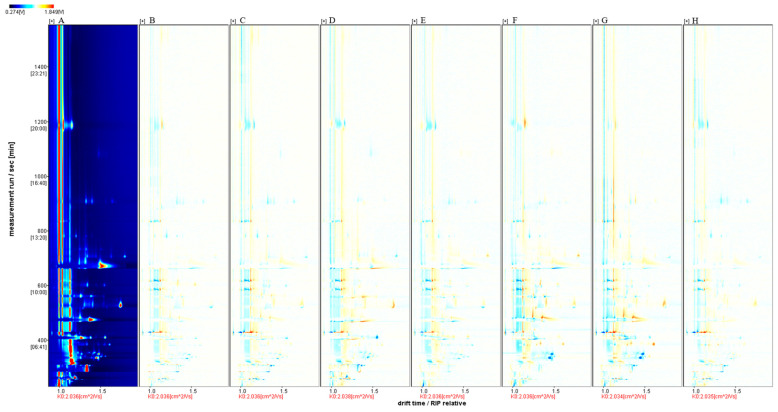
HS-GC-IMS difference spectrum of the sample. Note: Using A as a reference, the rest of the spectrum subtracts the signal peaks in A to obtain the difference spectrum between the two. Blue areas indicate less substance in this sample than in A. Red areas indicate more substance in this sample than in A. The darker the color, the more significant the difference. The difference between the samples can be seen from the above graph.

The ‘Beibinghong’ ice wine from the first treatment group was used as a reference, and the rest of the spectra were subtracted from the signal peaks in the first treatment to obtain the difference spectra (Figure 3).

### 2.4. Analysis of Volatile Components

Aroma is an essential index for evaluating the quality of wine, and the study of wine aroma mainly focuses on the aromatic substances that positively affect it. The critical role of SO_2_ in winemaking also includes its role in the aroma. SO_2_ also significantly affects the aroma of wine, with the addition of SO_2_ affecting the concentration of volatile compounds in the wine by between 33% and 43% [42]. Aromatic substances in wine can be categorized into terpenoids, aliphatic compounds, and aromatic compounds according to their chemical structure, of which aliphatic compounds include alcohols, acids, ketones, and esters. The analytical spectra and data were viewed with VOCal for qualitative and quantitative analysis, and the volatile components in the wine samples were characterized by the built-in NIST and IMS databases of HS-GC-IMS. A total of 44 typical volatile compounds were detected (Table 5), and the most significant number of species was 18 esters, 11 alcohols, 3 acids were detected, 5 ketones, and 11 uncharacterized volatile compounds. The volatile aroma compounds detected in the ice wine samples from the eight treatments were the same type, but the contents were significantly different. Among the eight treatments, the highest total volatile compound content was found in the seventh treatment group with 80 mg/L of added SO_2_, with a total volatile compound content of 81,930.42256 µg/L. The following treatments with the highest total volatile compound content in descending order were the treatment group with 50 mg/L of added SO_2_, with a volatile compound concentration of 81,394.73104 µg/L, treatment group with 70 mg/L SO_2_ addition 81,328.35368 µg/L, treatment group with 40 mg/L SO_2_ addition 81,182.37288 µg/L, treatment group with 60 mg/L SO_2_ addition 79,569.26936 µg/L, and treatment group with 90 mg/L SO_2_ addition 79,215.75989 µg/L. 79,569.26936 µg/L for 60 mg/L of SO_2_, 79,215.75984 µg/L for 90 mg/L of SO_2_, 78,921.88528 µg/L for 30 mg/L of SO_2_, and 77,818.71496 µg/L for 20 mg/L of SO_2_. Table 5 shows that alcohols accounted for the most significant proportion of 62.9–64.73%, followed by esters at 24–25.82%, and alcohols and esters were the main aroma compounds in the wine samples.

#### 2.4.1. Esters

The esters in wine are mainly produced by acyl-coenzyme A and fatty acids and alcohols in yeast cells under the catalytic action of relevant enzymes during alcoholic fermentation, and they have the aroma of fruits or flowers, which play a vital role in the aroma of wine [43,44]. Esters give wines a unique and complex fruity flavor, which is a critical component of their aroma composition, and the description of the aroma of the detected ester compounds also shows that the esters are mainly dominated by fruity aroma. The various ester compounds present in wines have coordinated compositional ratios and have synergistic effects on the formation of aroma [43,45]. The esters in grapes are mainly found in grape skins, which are fully macerated during fermentation, releasing the variety’s unique fruity and floral aromas. As can be seen in Table 4, esters were the compounds with the highest percentage of content in the assay, and from the esters detected, the esters that provided a higher concentration of aromas were isoamyl acetate, ethyl acetate, methyl formate, ethyl butyrate, and Ethyl propionate, the highest content of esters detected among the eight treatments was in treatment group 7 (SO_2_ addition of 80 mg/L) at 20,217.62344 µg/L, followed by ester volatile compounds in descending order by treatment group 8 (SO_2_ addition of 90 mg/L) at 20,201.48088 µg/L; treatment group 1 (SO_2_ addition of 20 mg/L) was 20,089.12584 µg/L; treatment group 5 (SO_2_ addition of 60 mg/L) was 19,966.58664 µg/L; treatment group 3 (SO_2_ addition of 40 mg/L) was 19,897.53192 µg/L; and treatment group 6 (SO_2_ addition of 70 mg/L) was 19,734.66768 µg/L; the fourth treatment group (SO_2_ addition of 50 mg/L) was 19,533.09736 µg/L; and the second treatment group (SO_2_ addition of 30 mg/L) was 19,438.08048 µg/L. A study by Teresa Garde-Cerdán et al. found [46] that when volatile aroma compounds were examined in wines with or without the addition of SO_2_, it was found that the concentration of SO2 did not have a significant effect on the total esters in the wines, which is the same as our findings, and this is probably because the wines are rich in unsaturated fatty acids. Therefore, the concentration of oxygen in the medium did not affect the formation of these compounds. Liu et al. [47] found that the addition of an appropriate concentration of SO_2_ can also increase the content of isoamyl acetate, ethyl acetate, and ethyl octanoate in wine to a certain extent, which can bring pleasant floral and fruity aroma to wine and increase the complexity of wine aroma. The high concentration of SO_2_ can lead to the increase of ethyl acetate, which can hurt the aroma quality of the wine. Therefore, when choosing the concentration of SO_2_, it is essential to consider not only the concentration of the aroma but also the negative effect of the aroma content on the wine, and the amount added should be manageable [42,48,49]. Moderate amounts of SO_2_ can help stabilize the aroma components in wine and contribute to forming certain aromas. However, excessive amounts of SO_2_ may react with the aroma components of the wine, resulting in a change or loss of aroma. Such changes may be manifested as a diminution or loss of certain aromas in the wine or the production of some unpleasant off-flavors.

#### 2.4.2. Alcohols

Alcohols accounted for the most significant percentage of 62.9–64.73% of the wines, and their aroma characteristics were mainly pungent or grassy. The content of alcohols between different treatment groups in descending order was 52,687.70472 µg/L in the fourth treatment group (SO_2_ addition of 50 mg/L); 52,419.32192 µg/L in the seventh treatment group (SO_2_ addition of 80 mg/L); 51,936.64448 µg/L in the sixth treatment group (SO_2_ addition of 70 mg/L); third treatment group (SO_2_ addition of 40 mg/L) at 51,900.99208 µg/L; fifth treatment group (SO_2_ addition of 60 mg/L) at 50,486.39344 µg/L; second treatment group (SO_2_ addition of 30 mg/L) at 50,107.1648 mg/L; the eighth treatment group (SO_2_ addition of 90 mg/L) with 50,067.50616 µg/L; and the first treatment group (SO_2_ addition of 20 mg/L) with 48,951.784 µg/L. The highest ethanol content was found in the treatment group with the highest SO_2_ addition of 70 mg/L, and the lowest was found in the treatment group with the lowest SO_2_ addition of 20 mg/L. The highest ethanol content was found in the treatment group with the highest SO_2_ addition of 70 mg/L and the lowest in the treatment group with the lowest SO_2_ addition of 20 mg/L. The highest ethanol content was in the treatment group with 70 mg/L of SO_2_, and the lowest was in the treatment group with 20 mg/L of SO_2_; the highest methanol content was in the treatment group with 50 mg/L of SO_2_, and the lowest methanol content was in the treatment group with 20 mg/L of SO_2_;

#### 2.4.3. Others

Aldehydes, acids, and ketones accounted for a relatively small percentage of the wine. The percentage of aldehydes in the volatile compounds of ice wine was 2.06–2.17%. Among the treatment groups, the most considerable aldehydes content was in the third treatment group (SO_2_ addition of 40 mg/L) with 1758.6688. The lowest content was in the eighth treatment group (SO_2_ addition of 90 mg/L) with 1627.96816 µg/L. The proportion of ketones in the volatile compounds of ice wine ranged from 1.87% to 2.02%, with the most considerable ketone content in the first treatment group (20 mg/L SO_2_) at 1575.7028 µg/L and the lowest in the fifth treatment group (60 mg/L SO_2_) at 1511.65784 µg/L. The proportion of acid compounds in the volatile compounds of ice wine ranged from 1.87% to 2.02%. The percentage of acid compounds in the volatile compounds of ice wine ranged from 4.73% to 5.66%, with the most significant amount of acid compounds in treatment group 6 (70 mg/L of SO_2_ addition) at 4604.20464 µg/L and the lowest in treatment group 1 (20 mg/L of SO_2_ addition) at 3%.

Among the treatments, regarding the total content of volatile compounds, the SO_2_ addition of 30 mg/L had a higher content of total aroma substances. Methanol and other substances harmful to the aroma components of ‘Beibinghong’ ice wine were fewer. The content of the components that impacted the quality was lower, so the quality of the wine was better than that of the other treatment groups from a comprehensive point of view.

### 2.5. Principal Component Analysis (PCA) of Wine Samples

**Figure 4:**
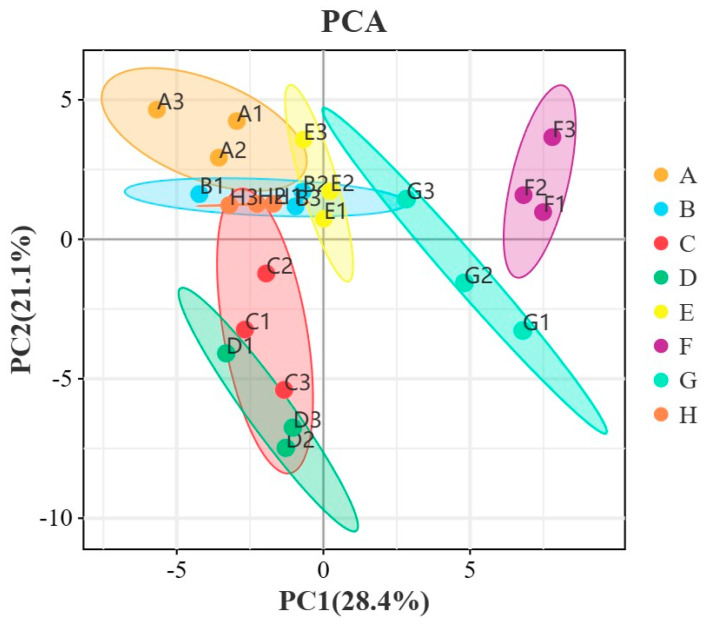
PCA analysis of the sample.

In order to better present and distinguish the differences between the different treatment ice wine samples, the known volatile compounds identified by GC-IMS were analyzed by PCA (Figure 4). Eight treatment groups of samples were well differentiated according to their aroma characteristics. PCA was performed on the samples to discriminate the magnitude of variability between the samples of the groups of different wines, between subgroups, and between samples within groups. The contribution rate of PC1 was 28.4%, and that of PC2 was 21.1%, and the eight groups of samples showed apparent separation trends on the two-dimensional graph, with no outlier samples. The samples of the same kind of wines were clustered well, with high experimental reproducibility. The PCA results show that the differences in aroma substances among the eight groups of samples are significant and clearly distinguishable from other samples. As shown in the figure, it can be seen that the four treatments of A, B, H, and E clustered together, the treatment groups of C and D clustered together, and the groups of G and F clustered together, which indicated that the volatile compounds were similar between these treatment groups. The concentration of the aroma compounds was also somewhat different between the different treatments.

#### 2.5.1. OAV Analysis of Major Aroma Compounds of Different Wine Samples

Generally, the components with OAV greater than one directly impacts the overall flavor and are the main components providing the flavor [50,51,52]. Based on the qualitative and quantitative results of GC–IMS, the threshold values of corresponding aroma compounds in water were found in the literature, and their OAV values were calculated. As shown in Table 6, a total of 21 aroma compounds with OAV values greater than one were detected in different ice wine samples, which were ethyl caprylate, 1-hexanol-M, ethyl caproate, 3-methyl-1-butanol, isoamyl acetate, hexanal, 2-methyl-1-propanol, ethyl butyrate, isobutyl acetate, pentanal, ethyl acrylate, ethyl isobutyrate, ethyl acetate, acetone, acetaldehyde, propionaldehyde, (Z)-3-hexenyl acetate, ethyl 3-methyl butanoate-M, ethyl 3-methyl butanoate-D, butyraldehyde, and 3-methyl butanal; studies have shown that the OAV values are directly proportional to the contribution of aroma [53,54]. Among the compounds with OAV values greater than 1, esters accounted for the most significant proportion of ten, and the OAV values of esters were significantly higher than those of other types of compounds, indicating that esters contribute to the prominent aroma of ‘Beibinghong’ ice wine. This is also in line with the GC–IMS results, where esters contributed the primary aromas in ‘Beibinghong’ ice wine, dominated by fruity and floral notes [55], followed by aldehydes, with six aldehydes having OAVs greater than 1, indicating that the grassy aroma of aldehydes is also a significant contributor to the aroma of ‘Beibinghong’ ice wine; there was also a ketone compound of acetone among the compounds with an OAV value of greater than 1; and there were three alcohols, which is also a significant contributor to the iconic wine flavor of the ‘Beibinghong’ ice wine aroma.

**Figure 5:**
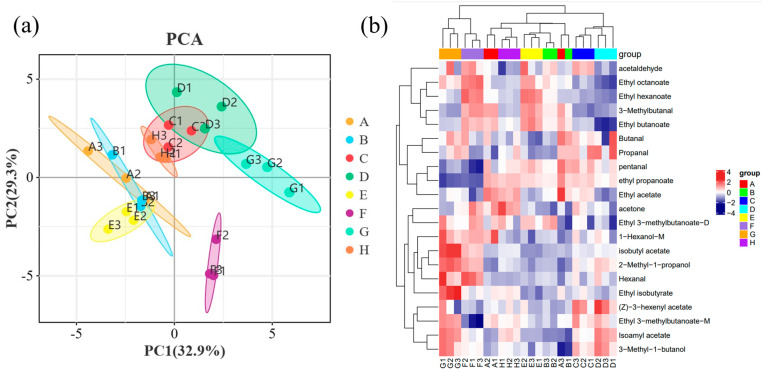
(**a**) PCA plots and (**b**) thermograms of volatile aroma compounds with OVA values greater than 1 in ice samples from different treatments of ‘Beibinghong’.

Principal component analysis (PCA) is a multivariate statistical analysis technique. Many complex and hard–to–find variables in the original sample are represented by identifying several principal component factors. The regularity and variability between samples are assessed based on the contribution of the principal component factors in different samples [56]. The PCA results clearly showed that two principal components were extracted from the PCA analysis of the concentration of volatile aroma compounds with OVA values greater than 1 in different treatments of ‘Beibinghong’ ice wine samples in a relatively independent space (Figure 5a), with a contribution of 32.9% for PC1 and 29.3% for PC2. Among the different varietal treatments, D, C, and H were located at the junction of one and four quadrants and were positive on PC2. G and D were located in the first quadrant of the score and were positive on PC1 and PC2. F was located in the second quadrant and was positive on PC1 and negative on PC2. E was located in the third quadrant and was negative on PC1 and PC2, and A and B were located in the junction of the third and fourth quadrants and were negative on PC1. This indicates significant differences in the volatile aroma compounds with OVA values greater than 1 in the ‘Beibinghong’ ice wine samples from different treatments. However, specific treatment groups also showed similarities in the aroma compounds with OAV values greater than 1.

Clustering of the concentration of volatile aroma compounds in nine sample wines with OVA values greater than 1. Based on the sample heat map analysis (Figure 5b), it was seen that the red color indicated that the aroma compound component was highly expressed in the sample, and the blue color indicated that the aroma compound was expressed at a lower level in the sample. The concentration of volatile aroma compounds with an OVA value of greater than 1 varied considerably among the samples of each variety. In general, most volatile aroma compounds with high aroma intensity values also have high OAV [57]; moreover, the two methods can be mutually verified. However, a small number of volatile aroma compounds also have low OAV despite high aroma intensity values, or vice versa. In this experiment, screening volatile aroma compounds between different treatment groups using OAV values can more accurately extract the critical volatile aroma compounds that may affect the aroma of ‘Beibinghong’ ice wine.

#### 2.5.2. Analysis of Volatile Compounds 0PLS-DA in Wine

**Figure 6:**
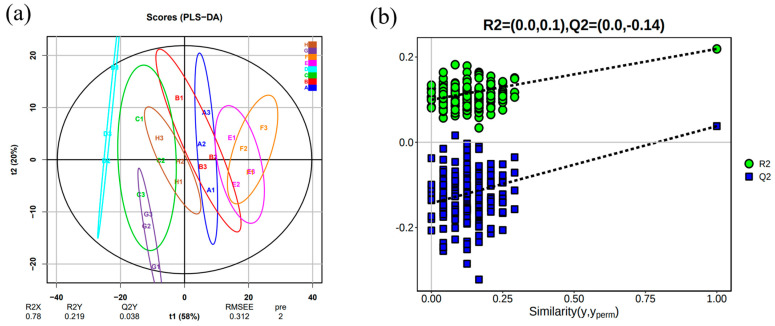
Analysis of different treatments of ‘Beibinghong’ ice wine samples OPLSA-DA ((**a**) Score chart of the OPLS-DA model of the sample, (**b**) Sample replacement test chart).

OPLS-DA is a statistical method for supervised discriminant analysis [54,58,59]. The contribution of each variable to the flavor of the wine was further quantified according to the variable important for the projection (VIP) in the OPLS-DA model [59]. OPLS-DA was validated with 200 permutations and found that R2 and Q2 were more significant than the model after Y replacement (Figure 6b). Thus, the model predictions were reliable, and variables with VIP > 1 could be used as potential biomarkers. This experiment used the OVA values of compounds with an OAV value greater than 1 in the composition of wine samples from different varieties as Y variables for OPLS-DA analysis.

Screening of compounds with VIP values > 1 as marker compounds for wine (Table 7). The results revealed that the compounds that may affect the aroma differences at different SO_2_ concentration treatments might be related to ethyl butyrate, ethyl propionate, ethyl 3-methyl butyrate-M, ethyl 3-methyl butyrate-D, and 3-methyl butyraldehyde.

### 2.6. Sensory Evaluation of ‘Beibinghong’ Ice Wine Brewed with Different Concentrations of SO_2_

Sensory evaluation is a crucial way for consumers to assess the quality of a wine [60]. Sensory evaluation of wines influences consumer choice. As seen in the sensory evaluation, it can be found (Table 8) that the color and clarity of wine samples from the eight treatment groups were above 9 points, and their color and clarity scores were the highest in the sixth treatment group, and the aroma and taste scores were the highest in the third treatment group; the typicality of the wines from the third and the sixth treatment groups had the highest scores; the highest total score was obtained from the third treatment group, and the lowest total score was obtained from the first treatment group. In summary, the overall flavor of the ‘Beibinghong’ ice wines was the highest when SO_2_ was added to the ice wines at an added amount of 30 mg/L. Adding too little or too much SO_2_ can affect the wine’s flavor and lead to certain defects. Pelonnier-Magimel E et al. [30] studied whether the Bordeaux quality wines without added SO_2_ have their typicality to assess the organoleptic specificity of the wines without added SO_2_. Finally, it was found that wines without added SO_2_ had a much higher frequency of defects than wines with added SO_2_ (70 percent and 15 percent, respectively). Therefore, the absence of SO_2_ in production can significantly impact the wine’s flavor. If the wine is to maintain its typicality in production, as little SO_2_ as possible can be added, while ensuring the flavor and bactericidal effect [34,61].

## 3. Conclusions

In this study, we investigated the effects of SO_2_ addition on the fermentation process and volatile aroma compounds of ‘Beibinghong’ ice wine. The basic physicochemical properties of ice wine and the types and contents of volatile aroma compounds were analyzed in different SO_2_ treatment groups, and the fingerprints of volatile compounds in ice wine brewed with different concentrations of SO_2_ were constructed. In this study, it was found that the fermentation time of ice wine was shortest and the total acid content was relatively low at 10 mg/L, but the types and contents of volatile aroma compounds did not increase significantly; the total content of volatile aroma compounds of ice wine was highest at 80 mg/L. OPLS-DA calculated the VIP values, and the joint analysis of OAV and VIP values further identified five compounds: ethyl butyrate, ethyl propionate, ethyl 3-methyl butyrate-M, ethyl 3-methyl butyrate-D, and 3-methyl butyraldehyde, which were significantly different among the groups treated with different concentrations of SO_2_. These compounds might be the critical factors of the effect of SO_2_ on the volatile aroma compounds of the ‘Beibinghong’ ice wine. These compounds may be critical factors in the effect of SO_2_ on the volatile aroma compounds of ‘Beibinghong’ ice wine. Tasting of ‘Beibinghong’ ice wine with different SO_2_ additions revealed that the overall flavor of ‘Beibinghong’ ice wine was the highest at 30 mg/L. In conclusion, the wine’s best accumulation of nutrients and flavor was achieved at 30 mg/L of SO_2_ additions. To some extent, this experiment reflects the differences in the quality of wines at different concentrations of SO_2_ through micro-winemaking, which provided a reference for the development and promotion of wines, and the results of the study provided a basis for optimizing the fermentation process of the ‘Beibinghong’ ice wine. As SO_2_ is a cheap and effective wine preservative, its complete replacement is not feasible. The optimum level of SO_2_ addition in the production of ‘Beibinghong’ ice wine was investigated in order to minimize the excessive use of SO_2_ in wine production. The results of this study provide some theoretical basis for optimizing the fermentation process of ‘Beibinghong’ ice wine and improving the quality of ice wine.

## Figures and Tables

**Table 1:** Gas chromatography conditions.

Time (Min:Sec)	E1	E2	Recording
00:00,000	150 mL/min	2 mL/min	rec
02:00,000	150 mL/min	2 mL/min	-
10:00,000	150 mL/min	10 mL/min	-
20:00,000	150 mL/min	100 mL/min	-
30:00,000	150 mL/min	100 mL/min	stop

**Table 2:** Sensory score.

Item	Percentage	Features	Full Marks
Color	10%	Chroma and hue	10
Clarification	10%	Degree of clarification	10
Aroma	30%	Finesse	5
		Richness	5
		Coherence	5
		Variety characteristics	5
		Duration	5
		Variation and complexity (multiple levels of aroma)	5
Taste	40%	Balance and coordination	10
		Body and fullness (weightiness in the mouth)	10
		Texture and structure	5
		Continuity and layers	5
		Flavor quality and persistence	5
		Lingering flavor	5
Typicality	10%	Synthesize and evaluate	10
Totals			100

**Table 3:** Fermentation process and basic physicochemical indexes of ‘Beibinghong’ ice wine with different SO_2_ additions.

SO_2_ Concentration (mg/L)	Start of Fermentation (h)	End of Fermentation (days)	Soluble Solids (%)	Total Sugar (g/L)	Total Acid (g/L)	Dry Extract (g/L)	Alcohol Content (*v*/*v*)
10	24 ± 0 f	23 ± 1 e	26.3 ± 0.5 b	162.66 ± 13.2 d	11.36 ± 0.41 bc	151.24 ± 15.61 d	11.5 ± 0.50 bc
20	72 ± 0 d	34 ± 3 d	26.5 ± 2.0 b	163.57 ± 8.06 c	11.28 ± 1.10 c	150.33 ± 7.58 e	11.5 ± 0.36 bc
30	48 ± 0 e	34 ± 3 d	25.4 ± 1.7 c	162.19 ± 5.13 d	11.26 ± 1.65 c	153.71 ± 6.42 b	12 ± 1.0 a
40	48 ± 0 e	23 ± 1 e	26.5 ± 1.0 b	160.96 ± 10.7 e	11.32 ± 0.26 bc	156.94 ± 13.33 a	11.5 ± 0.72 bc
50	48 ± 0 e	21 ± 2 f	26.7 ± 2.0 b	161.08 ± 4.5 e	11.48 ± 0.15 abc	152.82 ± 17.21 c	11.5 ± 0.21 bc
60	96 ± 0 c	35 ± 4 c	27.8 ± 1.0 a	164.69 ± 5.57 b	11.65 ± 1.03 abc	149.21 ± 4.31 f	11 ± 0.06 cd
80	168 ± 0 b	64 ± 9 a	28.3 ± 0.8 a	168.73 ± 11.02 a	12.06 ± 1.02 ab	145.17 ± 15.30 g	10.5 ± 0.42 d
100	192 ± 0 a	38 ± 5 b	28.5 ± 1.7 a	168.92 ± 19.20 a	12.15 ± 0.78 a	144.98 ± 7.06 g	10.5 ± 0.11 d

Means with different letters in the same column express significant differences (Duncan’s test *p* < 0.05).

**Table 4:** Content of anthocyanin in ‘Beibinghong’ ice wine with different SO_2_ additions.

SO_2_ (mg/L)	10	20	30	40	50	60	80	100
Total anthocyanin (µg/L)	126.63 ± 11.26 h	133.87 ± 7.48 f	155.86 ± 2.51 c	135.26 ± 9.06 e	140.27 ± 3.29 d	172.56 ± 11.38 b	194.26 ± 26.93 a	132.76 ± 18.71 g

Means with different letters in the same column express significant differences (Duncan’s test *p* < 0.05).

**Table 5:** Composition of volatile compounds in ‘Beibinghong’ ice wine treated with different concentrations of SO_2_.

Serial No.		Retention Time (sec)	Substances	Aroma Description	Substance Content (µg/L)							
					Sample No. 1	Sample No. 2	Sample No. 3	Sample No. 4	Sample No. 5	Sample No. 6	Sample No. 7	Sample No. 8
1		1080.89	Ethyl octanoate	Apricot, Brandy, Fat, Floral, Pineapple	322.722 ± 19.384	347.309 ± 13.027	296.373 ± 17.554	248.987 ± 13.003	376.587 ± 32.87	419.786 ± 8.103	360.532 ± 17.447	310.012 ± 6.096
2		886.48	Ethyl lactate-M	Cheese, Floral, Fruit, Pungent, Rubber	357.994 ± 50.581	330.748 ± 9.635	345.173 ± 6.24	393.777 ± 9.815	378.219 ± 11.958	302.744 ± 2.979	333.986 ± 37.971	318.373 ± 5.942
3		885.16	Ethyl lactate-D	Cheese, Floral, Fruit, Pungent, Rubber	41.248 ± 10.967	27.997 ± 2.465	30.767 ± 4.564	33.067 ± 6.445	28.271 ± 3.16	29.047 ± 5.952	31.753 ± 3.379	29.832 ± 3.881
4		707.83	Ethyl hexanoate	Cheese, Floral, Fruit, Pungent, Rubber	408.356 ± 25.405	414.364 ± 31.845	360.863 ± 18.269	322.325 ± 11.466	548.61 ± 17.016	555.558 ± 18.26	394.243 ± 29.17	382.963 ± 9.191
5		526.01	Isoamyl acetate	Apple, Banana, Pear	2867.294 ± 58.401	2775.802 ± 94.577	3217.441 ± 64.118	3539.672 ± 96.581	2949.724 ± 2.712	3181.046 ± 71.388	3621.83 ± 67.244	3319.403 ± 54.505
6		409.12	Ethyl butanoate	pineapple flavor	712.02 ± 17.695	653.213 ± 6.981	577.538 ± 7.574	417.204 ± 11.829	765.006 ± 29.341	725.577 ± 20.563	580.78 ± 6.748	617.956 ± 16.818
7		383.33	isobutyl acetate	Apple, Banana, Floral, Herb	266.451 ± 3.555	255.106 ± 3.543	297.941 ± 9.633	308.365 ± 5.877	224.468 ± 3.165	377.387 ± 14.584	512.87 ± 10.248	302.304 ± 2.072
8		350.14	propyl acetate	Celery, Floral, Pear, Red Fruit	589.059 ± 20.017	535.422 ± 7.295	571.309 ± 0.436	561.966 ± 4.026	554.909 ± 11.265	327.439 ± 5.638	295.958 ± 12.592	600.24 ± 9.152
9		336.31	ethyl propanoate	Apple, Pineapple, Rum, Strawberry	904.927 ± 50.425	783.426 ± 25.267	827.193 ± 14.304	760.554 ± 30.164	802.542 ± 14.539	479.4 ± 17.647	460.67 ± 32.085	845.108 ± 17.746
10		340.92	Ethyl isobutyrate	fruit	189.551 ± 5.722	173.682 ± 1.248	184.696 ± 6.095	174.112 ± 6.533	163.053 ± 6.197	187.054 ± 6.442	233.276 ± 8.061	192.519 ± 1.879
11		292.51	Ethyl acetate	Aromatic, Brandy, Grape	10,270.327 ± 79.702	10,043.96 ± 35.944	10,070.65 ± 23.923	9856.912 ± 39.493	9926.147 ± 32.15	9879.314 ± 112.062	9994.921 ± 63.219	10,158.871 ± 27.223
12		263.7	Ethyl formate	Pungent	2485.568 ± 36.304	2385.056 ± 11.981	2380.013 ± 13.785	2164.664 ± 5.431	2485.498 ± 36.882	2487.519 ± 7.555	2639.144 ± 16.111	2418.238 ± 6.044
13		732.02	Butyl butanoate	Apple, pineapple flavor	314.625 ± 20.591	339.229 ± 24.721	346.936 ± 5.068	377.007 ± 20.819	372.044 ± 14.43	362.305 ± 5.044	343.359 ± 12.26	331.742 ± 9.67
14		767.45	(Z)-3-Hexenyl acetate	Banana, floral	51.247 ± 2.123	50.296 ± 1.656	57.74 ± 4.47	60.325 ± 4.389	50.99 ± 3.059	49.451 ± 1.981	52.637 ± 7.373	52.258 ± 2.257
15		516.1	Butyl propionate	Fruit	88.274 ± 3.05	99.196 ± 7.891	98.357 ± 5.902	88.606 ± 7.85	100.681 ± 5.435	140.951 ± 4.581	114.616 ± 7.301	90.438 ± 1.154
16		424.71	Ethyl3-methylbutanoate-M	Apple, Mulberry Aroma	119.992 ± 2.944	113.215 ± 4.203	123.41 ± 5.895	121.37 ± 5.868	114.138 ± 3.706	98.191 ± 6.187	128.856 ± 1.569	119.214 ± 1.785
17		424.09	Ethyl3-methylbutanoate-D	Apple, Mulberry Aroma	63.356 ± 8.178	66.816 ± 14.996	61.928 ± 9.264	58.284 ± 8.347	70.701 ± 3.646	73.035 ± 5.266	71.903 ± 6.921	67.135 ± 8.382
18		444.3	Ethyl 2-methylbutanoate	Apple, Ester, Green Apple, Kiwi, Strawberry	36.115 ± 0.756	43.242 ± 5.611	49.203 ± 7.374	45.902 ± 3.926	54.997 ± 4.229	58.865 ± 4.477	46.289 ± 4.716	44.875 ± 2.32
No. of ester species	18	Total			20,089.12584	19,438.08048	19,897.53192	19,533.09736	19,966.58664	19,734.66768	20,217.62344	20,201.48088
		%			25.82	24.63	24.51	24	25.09	24.27	24.68	25.5
1		908.81	1-Hexanol-M	Banana, Flower, Grass, Herb	761.642 ± 160.852	664.524 ± 32.62	709.027 ± 50.629	695.845 ± 76.075	637.454 ± 59.986	802.564 ± 14.503	823.121 ± 75.245	650.641 ± 30.475
2		907.5	1-Hexanol-D	Banana, Flower, Grass, Herb	230.981 ± 77.158	172.311 ± 15.172	194.661 ± 16.176	197.904 ± 33.232	181.147 ± 34.356	246.892 ± 10.754	286.767 ± 50.847	173.555 ± 14.727
3		672.36	3-Methyl-1-butanol	brandy	11,466.999 ± 322.872	11,589.634 ± 344.147	12,161.029 ± 151.633	12,360.758 ± 264.311	11,737.75 ± 270.254	11,990.457 ± 38.299	12,405.587 ± 280.962	11,910.102 ± 94.116
4		560.94	1-Butanol	Fruit	566.218 ± 48.597	603.987 ± 42.987	747.541 ± 51.003	813.903 ± 68.365	606.207 ± 54.248	552.356 ± 4.052	549.573 ± 59.427	656.573 ± 8.621
5		472.65	2-Methyl-1-propanol	pungent odor	5164.863 ± 71.084	5345.103 ± 89.543	5632.972 ± 81.471	5768.918 ± 91.136	5318.293 ± 89.947	5916.619 ± 59.966	6204.353 ± 51.918	5508.228 ± 54.195
6		408.34	1-Propanol	Alcohol, Candy, Pungent	2975.777 ± 47.018	3039.996 ± 39.433	3196.466 ± 54.537	3380.095 ± 61.027	3033.742 ± 44.128	2616.758 ± 31.35	2550.967 ± 71.881	3153.482 ± 10.22
7		355.13	Iso-propanol	pungent odor	205.871 ± 14.473	209.079 ± 14.817	222.704 ± 19.452	202.013 ± 14.932	183.215 ± 10.323	187.335 ± 19.493	207.814 ± 30.254	204.116 ± 8.551
8		316.72	Ethanol	alcoholic flavor	27,126.098 ± 103.789	27,986.877 ± 144.839	28,515.642 ± 295.583	28,715.711 ± 181.66	28,281.016 ± 139.473	29,096.813 ± 127.829	28,853.397 ± 710.693	27,325.985 ± 86.132
9		304.42	Methanol	alcoholic flavor	226.817 ± 10.88	260.779 ± 9.167	270.235 ± 1.287	295.837 ± 5.807	277.732 ± 4.16	285.157 ± 7.154	288.702 ± 19.969	255.843 ± 5.281
10		856.39	3-methyl-1-pentanol	Fruit	19.148 ± 1.42	20.134 ± 0.737	24.437 ± 1.327	26.431 ± 2.355	20.43 ± 1.558	20.347 ± 1.472	22.287 ± 1.954	21.038 ± 0.308
11		734.1	1-Pentanol	alcoholic flavor	207.371 ± 15.316	214.742 ± 8.932	226.28 ± 9.042	230.289 ± 15.029	209.406 ± 6.591	221.346 ± 10.837	226.752 ± 13.725	207.941 ± 5.393
No. of alcohol species	11	Total			48,951.784	50,107.1648	51,900.99208	52,687.70472	50,486.39344	51,936.64448	52,419.32192	50,067.50616
		%			62.9	63.49	63.93	64.73	63.45	63.86	63.98	63.2
1		1184.66	Acetic acid-M	Acid, Fruit, Pungent, Sour, Vinegar	2054.497 ± 79.013	2128.91 ± 70.701	2107.461 ± 50.139	2023.938 ± 65.237	2100.214 ± 30.593	2214.206 ± 13.902	2077.372 ± 158.519	2037.589 ± 46.317
2		1187.29	Acetic acid-D	Acid, Fruit, Pungent, Sour, Vinegar	1451.248 ± 398.813	1903.677 ± 96.528	1755.035 ± 222.202	1736.671 ± 70.548	1608.712 ± 60.058	2177.888 ± 122.534	1989.475 ± 388.328	1604.389 ± 116.831
3		1528.26	Isobutyric acid	Burnt, Butter, Cheese, Sweat	175.997 ± 74.937	277.775 ± 5.854	311.54 ± 1.733	261.381 ± 20.018	246.378 ± 18.058	212.109 ± 35.442	179.723 ± 16.096	206.798 ± 15.601
No. of acid species	3	Total			3681.7424	4310.36144	4174.0356	4021.99056	3955.30352	4604.20464	4246.57128	3848.77752
		%			4.73	5.46	5.14	4.94	4.97	5.66	5.18	4.86
1		518.25	Hexanal	Apple, Fat, Fresh, Green, Oil	81.55 ± 4.566	87.068 ± 0.793	87.762 ± 6.23	90.012 ± 7.245	84.554 ± 2.101	108.163 ± 4.545	103.644 ± 10.978	82.262 ± 0.496
2		355.52	Pentanal	pungent odor	234.398 ± 19.219	230.222 ± 8.562	236.699 ± 6.672	225.716 ± 6.372	234.86 ± 3.745	193.785 ± 7.765	208.26 ± 7.021	227.682 ± 3.167
3		230.52	Acetaldehyde	Floral, Green Apple	591.445 ± 10.713	610.715 ± 20.006	638.589 ± 7.464	562.302 ± 17.218	614.15 ± 53.505	638.151 ± 24.136	609.98 ± 55.699	552.894 ± 24.171
4		265.32	Propanal	pungent odor	492 ± 27.458	491.237 ± 23.69	517.149 ± 20.744	526.488 ± 27.194	481.319 ± 11.761	489.426 ± 23.396	506.841 ± 16.626	504.963 ± 11.44
5		299.69	Butanal	lemon scent	89.624 ± 4.037	87.713 ± 3.454	89.178 ± 1.06	88.058 ± 6.826	83.719 ± 1.95	87.988 ± 3.23	89.737 ± 2.049	86.741 ± 1.879
6		1306.57	Benzaldehyde	Bitter Almond, Burnt Sugar, Cherry, Malt, Roasted Pepper	115.298 ± 12.179	136.866 ± 11.171	155.016 ± 16.2	136.091 ± 9.857	134.335 ± 5.562	139.781 ± 10.206	134.194 ± 7.058	129.152 ± 3.039
7		309.35	3-Methylbutanal	apple flavor	72.15 ± 0.211	57.976 ± 0.993	34.274 ± 3.734	38.078 ± 3.222	73.824 ± 4.135	73.536 ± 3.911	39.255 ± 3.173	44.275 ± 1.451
No. of aldehyde species	7	Total			1676.46248	1701.79744	1758.6688	1666.7448	1706.76016	1730.82896	1691.9112	1627.96816
		%			2.15	2.16	2.17	2.05	2.14	2.13	2.07	2.06
1		782.71	3-Hydroxy-2-butanone-M	Buttery	166.582 ± 8.812	151.271 ± 30.591	143.925 ± 20.161	144.25 ± 14.122	130.308 ± 3.805	128.757 ± 18.812	131.074 ± 31.528	125.517 ± 5.321
2		781.39	3-Hydroxy-2-butanone-D	Buttery	106.295 ± 15.869	97.755 ± 7.259	117.172 ± 15.478	133.046 ± 15.239	93.92 ± 9.742	93.471 ± 4.586	123.232 ± 21.554	102.916 ± 3.284
3		258.7	Acetone	Butter, Creamy, Green Pepper	1019.321 ± 12.794	988.156 ± 5.67	1014.408 ± 10.468	1002.652 ± 12.949	1002.012 ± 6.228	1025.531 ± 11.472	1002.946 ± 8.749	1035.26 ± 10.713
4		381.32	4-Methyl-2-pentanone	ketone odor	73.862 ± 2.163	79.874 ± 1.468	80.804 ± 1.759	88.907 ± 4.275	101.28 ± 4.113	103.437 ± 1.217	102.252 ± 2.736	104.469 ± 1.763
5		356.18	2-Pentanone	Fruit, Pungent	209.642 ± 4.206	202.415 ± 1.836	193.008 ± 4.084	160.568 ± 5.345	184.14 ± 2.373	170.299 ± 4.524	190.478 ± 3.963	204.548 ± 3.183
No. of ketone species	5	Total			1575.7028	1519.4704	1549.31728	1529.42328	1511.65784	1521.49704	1549.97976	1572.70792
		%			2.02	1.93	1.91	1.88	1.9	1.87	1.89	1.99
1		350.14	1		216.928 ± 6.115	233.045 ± 1.731	247.923 ± 7.816	247.363 ± 4.28	248.273 ± 5.335	202.989 ± 9.454	193.582 ± 8.193	233.392 ± 2.338
2		335.54	2		353.637 ± 9.007	379.626 ± 7.302	399.756 ± 16.515	395.541 ± 21.29	407.613 ± 1.878	375.575 ± 10.84	362.538 ± 14.597	379.164 ± 5.413
3		335.92	3		378.483 ± 11.402	377.544 ± 1.911	379.778 ± 14.451	386.267 ± 18.497	377.753 ± 17.064	302.47 ± 5.513	314.602 ± 16.398	385.479 ± 8.25
4		846.77	4		45.751 ± 10.74	39.73 ± 3.065	36.079 ± 1.483	42.046 ± 3.424	42.802 ± 1.533	41.685 ± 3.221	36.582 ± 8.861	42.381 ± 3.375
5		780.1	5		316.261 ± 31.987	277.719 ± 49.572	291.323 ± 53.825	315.245 ± 28.833	278.636 ± 8.194	252.474 ± 40.115	301.364 ± 77.458	253.721 ± 10.992
6		438.27	6		45.653 ± 2.394	46.319 ± 1.665	50.52 ± 3.754	49.329 ± 2.226	59.937 ± 0.194	78.5 ± 5.716	50.037 ± 0.974	50.825 ± 0.44
7		391.08	7		125.533 ± 8.327	133.177 ± 4.903	143.463 ± 11.165	141.022 ± 3.036	124.45 ± 2.218	133.933 ± 15.61	136.195 ± 8.149	130.383 ± 2.378
8		589.68	8		73.228 ± 2.132	77.55 ± 2.724	75.599 ± 3.334	74.943 ± 1.968	76.667 ± 0.702	84.907 ± 3.385	73.308 ± 3.223	72.36 ± 3.653
9		668.96	9		115.615 ± 1.826	120.402 ± 6.387	122.84 ± 4.661	123.177 ± 4.685	126.067 ± 4.301	128.344 ± 4.744	120.47 ± 5.171	119.037 ± 0.98
10		254.41	10		71.712 ± 6.866	66.46 ± 3.262	69.208 ± 3.714	67.92 ± 3.427	66.642 ± 1.678	69.151 ± 1.258	70.994 ± 2.404	71.019 ± 1.878
11		383.56	11		101.099 ± 13.283	93.438 ± 2.733	85.338 ± 5.32	112.917 ± 11.088	133.728 ± 7.189	130.483 ± 9.075	145.343 ± 8.451	159.559 ± 1.114
No. of other categories	11	Total			1843.89744	1845.01072	1901.8272	1955.77032	1942.56776	1800.51088	1805.01496	1897.3192
		%			2.37	2.34	2.34	2.4	2.44	2.26	2.2	2.4
**Total**					77,818.71496	78,921.88528	81,182.37288	81,394.73104	79,569.26936	81,328.35368	81,930.42256	79,215.75984

Note: Compound flavor description from the Flavornet database (https://www.femaflavor.org); http://www.flavornet.org; accessed on 6 June 2020.

**Table 6:** OAV analysis of major aroma compounds of ‘Beibinghong’ ice wine treated with different SO_2_ additions.

Serial No.	Substance	A	B	C	D	E	F	G	H
1	Ethyl octanoate	3.508 ± 0.211	3.775 ± 0.142	3.221 ± 0.191	2.706 ± 0.141	4.093 ± 0.357	4.563 ± 0.088	3.919 ± 0.19	3.37 ± 0.066
2	1-Hexanol-M	1.523 ± 0.322	1.329 ± 0.065	1.418 ± 0.101	1.392 ± 0.152	1.275 ± 0.12	1.605 ± 0.029	1.646 ± 0.15	1.301 ± 0.061
3	Ethyl hexanoate	81.671 ± 5.081	82.873 ± 6.369	72.173 ± 3.654	64.465 ± 2.293	109.722 ± 3.403	111.112 ± 3.652	78.849 ± 5.834	76.593 ± 1.838
4	3-Methyl-1-butanol	52.123 ± 1.468	52.68 ± 1.564	55.277 ± 0.689	56.185 ± 1.201	53.353 ± 1.228	54.502 ± 0.174	56.389 ± 1.277	54.137 ± 0.428
5	Isoamyl acetate	7.168 ± 0.146	6.94 ± 0.236	8.044 ± 0.16	8.849 ± 0.241	7.374 ± 0.007	7.953 ± 0.178	9.055 ± 0.168	8.299 ± 0.136
6	Hexanal	16.31 ± 0.913	17.414 ± 0.159	17.552 ± 1.246	18.002 ± 1.449	16.911 ± 0.42	19.817 ± 3.039	20.729 ± 2.196	16.452 ± 0.099
7	2-Methyl-1-propanol	5.165 ± 0.071	5.345 ± 0.09	5.633 ± 0.081	5.769 ± 0.091	5.318 ± 0.09	5.696 ± 0.419	6.204 ± 0.052	5.508 ± 0.054
8	Ethyl butanoate	791.133 ± 19.661	725.792 ± 7.757	641.709 ± 8.416	463.56 ± 13.144	850.006 ± 32.601	838.845 ± 41.953	645.312 ± 7.497	686.617 ± 18.687
9	isobutyl acetate	10.658 ± 0.142	10.204 ± 0.142	11.918 ± 0.385	12.335 ± 0.235	8.979 ± 0.127	13.266 ± 3.644	20.515 ± 0.41	12.092 ± 0.083
10	Pentanal	19.533 ± 1.602	19.185 ± 0.714	19.725 ± 0.556	18.81 ± 0.531	19.572 ± 0.312	17.49 ± 2.186	17.355 ± 0.585	18.974 ± 0.264
11	Ethyl propanoate	90.493 ± 5.042	78.343 ± 2.527	82.719 ± 1.43	76.055 ± 3.016	80.254 ± 1.454	59.23 ± 19.215	46.067 ± 3.208	84.511 ± 1.775
12	Ethyl isobutyrate	12.637 ± 0.381	11.579 ± 0.083	12.313 ± 0.406	11.607 ± 0.436	10.87 ± 0.413	12.232 ± 0.841	15.552 ± 0.537	12.835 ± 0.125
13	Ethyl acetate	2.054 ± 0.016	2.009 ± 0.007	2.014 ± 0.005	1.971 ± 0.008	1.985 ± 0.006	1.984 ± 0.012	1.999 ± 0.013	2.032 ± 0.005
14	Acetone	1.225 ± 0.015	1.188 ± 0.007	1.219 ± 0.013	1.205 ± 0.016	1.204 ± 0.007	1.221 ± 0.011	1.205 ± 0.011	1.244 ± 0.013
15	Acetaldehyde	23.658 ± 0.429	24.429 ± 0.8	25.544 ± 0.299	22.492 ± 0.689	24.566 ± 2.14	24.719 ± 1.073	24.399 ± 2.228	22.116 ± 0.967
16	Propanal	6.074 ± 0.339	6.065 ± 0.292	6.385 ± 0.256	6.5 ± 0.336	5.942 ± 0.145	6.029 ± 0.301	6.257 ± 0.205	6.234 ± 0.141
17	(Z)-3-hexenyl acetate	1.653 ± 0.068	1.622 ± 0.053	1.863 ± 0.144	1.946 ± 0.142	1.645 ± 0.099	1.584 ± 0.059	1.698 ± 0.238	1.686 ± 0.073
18	Ethyl 3-methylbutanoate-M	1199.918 ± 29.439	1132.15 ± 42.03	1234.101 ± 58.948	1213.696 ± 58.676	1141.382 ± 37.058	1041.044 ± 103.454	1288.565 ± 15.688	1192.138 ± 17.852
19	Ethyl 3-methylbutanoate-D	633.563 ± 81.778	668.157 ± 149.962	619.284 ± 92.636	582.841 ± 83.472	707.005 ± 36.459	716.13 ± 41.233	719.029 ± 69.215	671.354 ± 83.818
20	Butanal	5.637 ± 0.254	5.517 ± 0.217	5.609 ± 0.067	5.538 ± 0.429	5.265 ± 0.123	5.451 ± 0.282	5.644 ± 0.129	5.455 ± 0.118
21	3-Methylbutanal	180.374 ± 0.528	144.94 ± 2.482	85.685 ± 9.335	95.195 ± 8.055	184.561 ± 10.337	185.264 ± 11.209	98.137 ± 7.933	110.689 ± 3.628

**Table 7:** Analysis of VIP values of aroma compounds in different treatments of ‘Beibinghong’.

Substances	VIP Value
Ethyl butanoate	2.741464
ethyl propanoate	1.077886
Ethyl 3-methylbutanoate-M	2.23449
Ethyl 3-methylbutanoate-D	1.761919
3-Methylbutanal	1.550059

**Table 8:** Sensory score.

Item	Percentage	A	B	C	D	E	F	G	H
Color	10%	9.0 ± 0.06	9.1 ± 0.1	9.7 ± 0.15	9.2 ± 0.06	9.3 ± 0.21	9.9 ± 0.15	9.8 ± 0.10	9.7 ± 0.10
Clarification	10%	9.9 ± 0.06	10 ± 0	10 ± 0	10 ± 0	10 ± 0	10 ± 0	10 ± 0	10 ± 0
Aroma	30%	26.4 ± 1.02	27.2 ± 1.25	29.3 ± 0.67	28.4 ± 1.02	28 ± 0.78	27 ± 0.21	26.7 ± 0.53	26.1 ± 1.02
Taste	40%	36.1 ± 0.17	36.4 ± 2.08	39 ± 0.15	37 ± 1.0	38.1 ± 0.06	37.4 ± 1.03	38 ± 0.57	35.9±0.26
Typicality	10%	9.4 ± 0.15	9.5 ± 1.21	10 ± 0.06	9.8 ± 1.15	9.6 ± 0.27	10 ± 0	9.4 ± 0.21	9.7 ± 0.06
Totals	100%	90.7 ± 1.20	92.2 ± 1.27	98 ± 1.0	94.4 ± 0.57	95 ± 1.0	94.3 ± 2.07	93.9 ± 1.52	91.4 ± 0.70

## Data Availability

The original contributions presented in the study are included in the article, further inquiries can be directed to the corresponding author.
